# Combination Therapy with EpCAM-CAR-NK-92 Cells and Regorafenib against Human Colorectal Cancer Models

**DOI:** 10.1155/2018/4263520

**Published:** 2018-10-15

**Authors:** Qing Zhang, Haixu Zhang, Jiage Ding, Hongyan Liu, Huizhong Li, Hailong Li, Mengmeng Lu, Yangna Miao, Liantao Li, Junnian Zheng

**Affiliations:** ^1^Cancer Institute, Xuzhou Medical University, Xuzhou, Jiangsu 221002, China; ^2^The Affiliated Aoyang Hospital of Jiangsu University, Zhangjiagang, Jiangsu 215600, China; ^3^Jiangsu Center for the Collaboration and Innovation of Cancer Biotherapy, Xuzhou Medical University, Xuzhou, Jiangsu 221002, China

## Abstract

Adoptive chimeric antigen receptor-modified T or NK cells (CAR-T or CAR-NK) offer new options for cancer treatment. CAR-T therapy has achieved encouraging breakthroughs in the treatment of hematological malignancies. However, their therapeutic efficacy against solid tumors is limited. New regimens, including combinations with chemical drugs, need to be studied to enhance the therapeutic efficacy of CAR-T or NK cells for solid tumors. An epithelial cell adhesion molecule- (EpCAM-) specific second-generation CAR was constructed and transduced into NK-92 cells by lentiviral vectors. Immune effects, including cytokine release and cytotoxicity of the CAR-NK-92 cells against EpCAM-positive colon cancer cells, were evaluated *in vitro*. Synergistic effects of regorafenib and CAR-NK-92 cells were analyzed in a mouse model with human colorectal cancer xenografts. The CAR-NK-92 cells can specifically recognize EpCAM-positive colorectal cancer cells and release cytokines, including IFN-*γ*, perforin, and granzyme B, and show specific cytotoxicity *in vitro*. The growth suppression efficacy of combination therapy with regorafenib and CAR-NK-92 cells on established EpCAM-positive tumor xenografts was more significant than that of monotherapy with CAR-NK-92 cells or regorafenib. Our results provided a novel strategy to treat colorectal cancer and enhance the therapeutic efficacy of CAR-modified immune effector cells for solid tumors.

## 1. Introduction

Colorectal cancer (CRC) is one of the most common malignancies. CRC has reportedly become the fourth leading cause of cancer death worldwide [1]. The therapeutic approaches for colorectal cancer include surgery, chemotherapy, radiation therapy, and immunotherapy. Surgery is the primary therapeutic modality for this disease. Although chemotherapy is regarded as a primary strategy to inhibit tumor growth or metastasis, patients with advanced diseases or cancer recurrence after surgery remain difficult to cure [2].

Chimeric antigen receptor-modified T cell (CAR-T) therapy is a newly developed adoptive treatment for cancer. The treatment has been proven effective against hematological malignancies and showed strong efficacy in some solid tumors, such as metastatic neuroblastoma [3, 4], recurrent glioblastoma [5], and prostate cancer [6]. However, its therapeutic efficacy in other solid tumors, including CRC, is less impressive.

Several groups have reported studies treating CRC using CAR-T cells. Tamada et al. developed anti-fluorescein-5-isothiocyanate (FITC) CAR-T cells and used the anti-FITC CAR-T cells plus FITC-labeled cetuximab to treat mice with epidermal growth factor receptor- (EGFR-) positive CRC [7]. Schlimper et al. engineered cytokine-induced killer (CIK) cells with CAR targeting carcinoembryonic antigen (CEA) using blood lymphocytes from CRC patients. The CAR-modified CIK cells showed improved selectivity in targeting autologous CRC cells [8]. Daly et al. developed CAR-T cells with a chimeric receptor recognizing the colorectal cancer-associated antigen EGP40 [9]. Ang et al. developed EpCAM-specific CAR-T cells and used the CAR-T cells to treat mice with colorectal cancer xenografts [10]. In addition to these preclinical studies, four groups carried out clinical trials in which CRC was treated with CAR-T cells targeting HER2 [11], TAG-72 [12], CEA [13], or CEACAM5 [14]. Unfortunately, the patient that received HER2-specific CAR-T cells died of CAR-T-related toxicity, and CAR-T cells targeting the other three antigens did not show objective therapeutic effects.

EpCAM (CD326), a 40 kDa transmembrane glycoprotein, is overexpressed in many solid tumors, while it shows variable but generally low levels in the normal epithelium. For example, high expression of EpCAM was detected in 97.7% patients with colorectal adenocarcinoma [15]. Overexpression of EpCAM results in highly upregulated oncogene expression and promotes cell proliferation [16]. With the development of molecular technologies, EpCAM has gained attention as a potential target for diagnostic and antibody-based immunotherapies for a spectrum of malignancies [17]. The treatment improved the survival rate by 30% and decreased the recurrence rate by 27% over 7 years of evaluation [16]. Nonetheless, the treatment effects were limited. Hence, there is an urgent need for identification of new treatment options with improved therapeutic potential.

Natural killer (NK) cells are one type of innate immune effector cells that contribute to the body's immune defense. NK cells are capable of killing tumor cells, as well as producing cytokines without previous stimulation [18]. The NK-92 cell line was established from a 50-year-old male patient with rapidly progressive non-Hodgkin's lymphoma by Gong et al. and displays characteristics of activated NK cells [19]. Several theoretical advantages make NK-92 cells a potential therapeutic cell line for cancer. First, NK-92 cells lack expression of inhibitory killer Ig-like receptors (iKIRs). Second, NK-92 cells lack immunogenicity and do not cause graft-versus-host disease (GVHD). Third, NK-92 cells are easy to expand and available as an “off-the-shelf” product, which can greatly reduce the treatment cycle and the cost of treatment [20]. General safety of infused NK-92 cells has been established in phase I clinical trials with clinical response observed in some treated renal cancer patients [21]. To enhance their therapeutic efficacy, NK-92 cells have been modified to express CARs against different cancer targets, including CD20 for lymphoma and leukemia, CD19 for chronic lymphocytic leukemia (CLL), GD2 for neuroblastoma, and EpCAM for breast carcinoma [22].

The hostile microenvironment composed of immunosuppressive cells, including carcinoma-associated fibroblasts (CAFs), and cytokines, including platelet-derived growth factor (PDGF), significantly impedes the efficacy of immunotherapeutic approaches. The mechanisms by which these factors suppress the immune system have been well defined in previous reviews [23–25]. Elimination or inhibition of these immunosuppressive factors will significantly promote an antitumor immune response and enhance the response to CAR-T or CAR-NK therapy. Treatment with chemotherapeutic agents may be a promising strategy to remodel the immunosuppressive tumor microenvironment and facilitate CAR-T or CAR-NK therapy. Combination therapy of CAR-T or CAR-NK with approved chemotherapeutic agents is convenient for clinical application [26].

Regorafenib is a potent multikinase inhibitor with activity against a range of protein kinases involved in oncogenesis (KIT, RET, and RAF), angiogenesis (VEGFR1–3 and TIE2), and maintenance of the tumor microenvironment (PDGFR and FGFR) [27]. Regorafenib has been recently approved for the treatment of metastatic CRC and gastrointestinal stromal tumors. Its antitumor activity has been demonstrated in a variety of preclinical models and is associated with suppression of cell proliferation, induction of apoptosis, and inhibition of tumor angiogenesis. Although regorafenib significantly prolongs overall survival in patients with metastatic CRC, its overall clinical efficacy remains quite limited [28].

A number of recent studies have indicated that besides their direct tumoricidal activity, some tyrosine kinase inhibitors (TKIs) can also modulate the tumor microenvironment and promote antitumor immunity. Doxorubicin [29, 30], sunitinib [31, 32], sorafenib [33, 34], and gemcitabine [35–37] have been shown to remodel the immune suppressive microenvironment and enhance the antitumor immune response. They can augment the therapeutic efficacy of immunotherapy through combined application with them [30, 35, 38–40]. Regorafenib is a sorafenib-related compound [41]. Although there is no previous report showing the immunomodulatory function of regorafenib, in this study, we constructed a second-generation CAR against EpCAM and sought to investigate whether combination therapy with the CAR-NK cells and regorafenib can enhance anticancer activity against human CRC in a mouse model.

## 2. Materials and Methods

### 2.1. Cell Lines

Human colorectal cancer cell lines (HCT116 and HCT-8) as well as the human embryonic kidney epithelial cell line 293T were cultured in Dulbecco's modified Eagle's medium (DMEM, Gibco, Life Technologies, America). The human colonic epithelial cell line FHC was cultured in RPMI-1640 medium (Gibco, Life Technologies, America). The human colorectal cancer cell line SW620 was cultured in Leibovitz's L-15 medium (Gibco, Life Technologies, America). All the above media were supplemented with 10% FBS and 1% penicillin/streptomycin (Gibco, Life Technologies, America). NK-92, CAR-NK-92, and Ctrl-NK-92 cells were incubated in alpha modification of Eagle's minimum essential medium (*α*-MEM, Gibco, Life Technologies, America) supplemented with 2 mM L-glutamine, 0.2 mM myo-inositol, 0.02 mM folic acid, 0.1 mM 2-mercaptoethanol, 400 IU/ml IL-2 (PeproTech, America), 12.5% FBS, 12.5% horse serum (Gibco, Life Technologies, America), and 1% penicillin/streptomycin. All cell lines were cultured at 37°C in a humidified atmosphere with 5% CO_2_.

### 2.2. Construction of EpCAM-CAR and Lentivirus Preparation

The EpCAM-CAR construct sequentially contains a mouse anti-human EpCAM scFv fragment (MOC31), CD8 hinge and transmembrane domains, and the intracellular signaling domains of 4-1BB and CD3*ζ*. The DNA sequence of the EpCAM-CAR was synthesized by GenScript (Nanjing, China). Then, the DNA fragment was inserted into a lentiviral vector-designated pRRL-GFP to generate the pRRL-EpCAM-CAR-GFP construct. The EpCAM-CAR and GFP were connected by a p2A peptide. To produce lentiviral particles, the plasmid pRRL-EpCAM-CAR-GFP was transduced into 293T cells with Δ809 and VSVG. Forty-eight hours after transfection, viral supernatant was harvested and spun at 4°C and 2000 rpm for 7 min. The viral supernatant then was filtered through a 0.45 *μ*m filter. The filtered supernatant was spun at 4°C and 25000 rpm for 2 hours. The supernatant was discarded. The pellet was resuspended in base medium with a volume of 1/100 the initial viral supernatant. The virus was aliquoted and stored at −80°C.

### 2.3. Transduction of NK-92 Cells

For lentivirus infection, a 24-well plate was coated with RetroNectin (TaKaRa, Shiga, Otsu) overnight at 4°C, according to the manufacturer's protocol, and then blocked with 2% BSA at room temperature for 30 min. Next, 2 × 10^5^ NK-92 cells were inoculated into 24-well plates, and lentivirus (MOI = 5) and polybrene at a final concentration of 8 μg/ml were added into the well. The plate was centrifuged at 32°C and 1500*g* for 1.5 hours. After overnight culturing, the infection protocol was repeated again. After expansion for several days, GFP+ NK-92 cells were sorted with a FACS system (FACSAria III, Becton-Dickinson, USA).

### 2.4. Flow Cytometric Analysis

For analysis of the lentivirus transduction rate of NK-92 cells, the GFP expression levels in Ctrl-NK-92 (control lentivirus with GFP-infected NK-92 cells) and CAR-NK-92 were analyzed by a FACS system (FACSCanto II, Becton-Dickinson, USA). For analysis of EpCAM surface expression, 1 × 10^6^ cancer cells were incubated with FITC-labeled mouse anti-human EpCAM antibody (324204, BioLegend) or isotype control (400310, BioLegend) in 200 *μ*l phosphate-buffered saline (PBS) with 2% bovine serum albumin (BSA) for 30 min at room temperature in dark, washed, and then analyzed by the FACS system (FACSCanto II, Becton-Dickinson, USA).

### 2.5. Western Blot Analysis

Whole-cell lysates were prepared with RIPA buffer (Beyotime Biotechnology) with 0.1 mM phenylmethylsulfonyl fluoride (PMSF, Sigma). Equal amounts of cell lysates (25 *μ*g) were loaded and separated on 10% SDS-PAGE and transferred onto a pure nitrocellulose blotting membrane (Amersham, Sweden). After the membranes were blocked, they were incubated with a primary rabbit anti-human anti-CD3*ζ* antibody (1 : 1000; ab40804, Abcam) or rabbit anti-human GAPDH antibody (1 : 1000; GTX100118, GeneTex). The membranes were then incubated with a horseradish peroxidase-conjugated anti-rabbit IgG. Target proteins were detected by the ECL system (Millipore) and visualized with the ChemiDoc XRS system (Bio-Rad).

### 2.6. Cytokine Release Analysis by ELISA

First, 1 × 10^4^ target cells were cocultured with effector cells at an effector cell : target cell (E : T) ratio of 2 : 1 in round-bottom 96-well culture plates for 24 h. Cell-free supernatants were assayed for cytokine secretion by enzyme-linked immunosorbent assay (ELISA) kits according to the manufacturer's protocol. Human IFN-*γ* and perforin ELISA kits were purchased from Dakewe Biotech Company. Human granzyme B ELISA kits were purchased from BioLegend.

### 2.7. Cytotoxicity by LDH Release Assay

1 × 10^4^ target cells were cocultured with CAR-NK-92 or Ctrl-NK-92 cells at E/T ratios of 1 : 1, 5 : 1, 10 : 1, 20 : 1, or 40 : 1 in RPMI-1640 with 15 mM HEPES and 5% FBS for 4 h. Released lactate dehydrogenase (LDH) in supernatants was measured using a CytoTox 96 Nonradioactive Cytotoxicity Assay Kit (Promega, Madison, WI, USA) according to the manufacturer's instructions. Specific cytotoxicity was calculated according to the following formula: % cytotoxicity = 100 × [(experimental release − effector spontaneous release − target spontaneous release)/(target maximal release − target spontaneous release)].

### 2.8. In Vivo Efficacy Studies

The local committee for animal care approved all animal studies. Six-week-old female NOD/SCID mice were purchased from Beijing Vital River Laboratory Animal Technology Co., Ltd. First, 3 × 10^6^ HCT-8 cells overexpressing luciferase (HCT-8-Luc) in 100 *μ*l DMEM medium were subcutaneously injected into the right flank of every mouse. When tumors grew to a palpable size below the skin surface (day 14), mice were randomly assigned to five groups (*n* = 6) and treated with the following regimens: vehicle alone (PEG400/125 mM aqueous methanesulfonic acid (80/20)), regorafenib (10 mg/kg, by gavage, 5 days per week for 4 weeks), EpCAM-CAR-NK92/Ctrl-NK-92 cells (1 × 10^7^ cells, by i.v. injection, once per week for 4 weeks), and a combination of regorafenib with EpCAM-CAR-NK-92 cells. Meanwhile, the mice that received NK cell therapy received 2000 IU recombinant human IL-2 (rhIL-2) by intraperitoneal injection every day. The length and width of the tumor were measured using a digital caliper, and the volume of the tumor was calculated using the following formula: tumor volume = length × width^2^/2. At the end of the experiment, tumor size was also monitored by *in vivo* bioluminescent imaging (BLI). Then, the mice were sacrificed, and tumors were harvested.

### 2.9. In Vivo Persistence Assay of NK-92 Cells

For persistence of NK-92 cells in the blood, on days 15, 21, and 31, 50 *μ*l blood was collected from the tail vein, and cell pellets were isolated by centrifugation at 5000 rpm for 5 minutes. Red blood cells were lysed using ACK lysis buffer (Gibco, CAT number A1049201) at room temperature for 5 minutes. Cells were then resuspended in FACS buffer, and surface markers were stained with anti-hCD45 (304014, BioLegend) and anti-hCD56 (362504, BioLegend) antibodies followed by flow cytometry analysis. For persistence of NK-92 cells in tumors, after treatment for 28 days (day 42), tumors were harvested following euthanasia. Tumor tissues were cut into small pieces. Single-cell suspensions were obtained by processing the small pieces of tumor tissues with a Gentle MACS Dissociator (Miltenyi Biotec, Germany) and passing cells through 70 *μ*m filter. Then, 2 × 10^6^ cells of each tumor were stained with the anti-hCD45 and anti-hCD56 antibodies and analyzed by a FACS system (FACS Canto II, Becton-Dickinson, USA).

### 2.10. Statistical Analysis

The data were analyzed using GraphPad Prism 5 software and are presented as the mean ± SEM. Statistical differences between the results of two groups were evaluated using a two-tailed Student's *t*-test. The differences with *p* < 0.05 were considered statistically significant (^∗^*p* < 0.05; ^∗∗^*p* < 0.01; ^∗∗∗^*p* < 0.001).

## 3. Results

### 3.1. Preparation and Characterization of EpCAM-Specific CAR-NK-92 Cells

**Figure 1:**
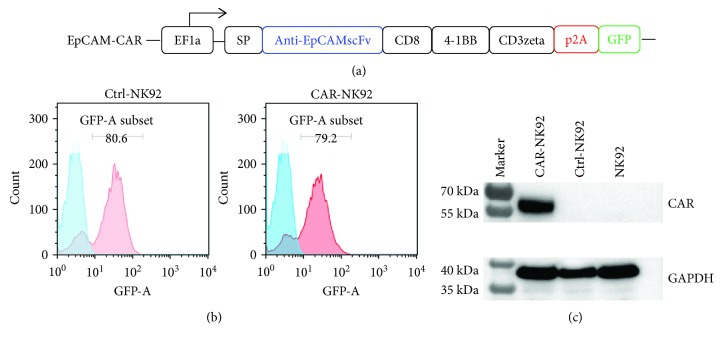
Generation and characterization of EpCAM-specific CAR-NK-92 cells. (a) Structure diagram of EpCAM-specific CAR. EF1*α*, promoter; SP, signal peptide; scFv, single-chain variable fragment. (b) Transduction efficiency of lentivirus in NK-92 cells. NK-92 cells were transduced with empty lentivirus vector (Ctrl-NK-92) or lentivirus containing the EpCAM-specific CAR encoding sequence (CAR-NK-92) and sorted by a FACS machine with a GFP marker. The GFP-positive rate of transduced NK-92 cells after sorting is shown. (c) Western blot analysis of the CAR expression in NK-92 cells with a monoclonal anti-human CD3*ζ* antibody. Glyceraldehyde-3-phosphate dehydrogenase (GAPDH) was also detected as an internal control.

A second-generation CAR, consisting of EpCAM-specific scFv linked to a CD8 hinge and transmembrane domains and the intracellular signaling domains of 4-1BB and CD3*ζ* in sequence (Figure 1(a)), was constructed and inserted into a lentiviral vector system with sequences encoding green fluorescent protein (GFP). The NK-92 cell line was transduced with the EpCAM-specific CAR and empty lentiviral vector to generate CAR-NK-92 and Ctrl-NK-92 cells, respectively. As shown in Figure 1(b), after FACS sorting of the transduced NK-92 cells with the GFP marker, the proportions of GFP-positive cells in both CAR- and empty vector-transduced NK-92 cells were approximately 80%. To validate expression of EpCAM-CAR in transduced NK-92 cells, we performed Western blot analysis using a rabbit anti-human CD3*ζ* monoclonal antibody that recognized the *ζ* chain portion of human CD3. As shown in Figure 1(c), the EpCAM-CAR was only detected at approximately 55 kDa in the CAR-transduced NK-92 cells.

### 3.2. Cytokine Release of EpCAM-Specific CAR-NK-92 Cells In Vitro

**Figure 2:**
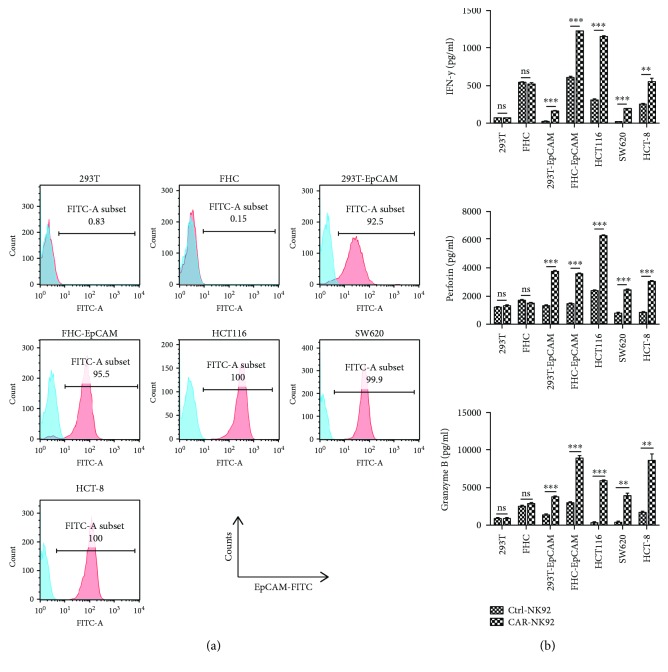
Specific cytokine release of EpCAM-specific CAR-NK-92 cells against EpCAM-positive cells. (a) FACS was used to test the surface expression of EpCAM proteins in 293T, 293T-EpCAM, FHC, and FHC-EpCAM cells and the human colorectal cancer cell lines HCT116, SW620, and HCT-8. (b) The levels of cytokines, released by Ctrl-NK-92 and CAR-NK-92 cells, were measured by enzyme-linked immunosorbent assay (ELISA) after incubation for 24 h with EpCAM-negative or EpCAM-positive target cells at an effector-to-target (E/T) ratio of 2 : 1. ^∗∗^*p* < 0.01; ^∗∗∗^*p* < 0.001; ns: not significant.

To investigate the functions of the EpCAM-specific CAR-NK-92 cells, we constructed two cell lines overexpressing human EpCAM using the human embryonic kidney epithelial cell line 293T and the human colonic epithelial cell line FHC, named 293T-EpCAM and FHC-EpCAM, respectively. FACS was used to assess the surface expression of EpCAM in 293T, 293T-EpCAM, FHC, FHC-EpCAM, and human colorectal cancer cell lines, including HCT116, SW620, and HCT-8. EpCAM was strongly expressed in 293T-EpCAM, FHC-EpCAM, and all three colorectal cancer cell lines but was absent in the 293T and FHC cell lines (Figure 2(a)).

To investigate whether the CAR-NK-92 cells could specifically recognize and be activated by EpCAM-positive cells, cytokine release assays were performed. The CAR-NK-92 and Ctrl-NK-92 cells were cocultured with target cells for 24 h at an E : T ratio of 2 : 1. After the incubation, levels of cytokines released by CAR-NK-92 cells, including IFN-*γ*, perforin, and granzyme B, were significantly elevated in the supernatants of EpCAM-positive 293T-EpCAM, FHC-EpCAM, HCT116, SW620, and HCT-8 cells compared with those of Ctrl-NK-92 cells. However, the levels of cytokines released by CAR-NK-92 cells and Ctrl-NK-92 cells were comparable when they were cocultured with EpCAM-negative 293T and FHC cells (Figure 2(b)). These results indicate that the CAR-NK-92 cells can specifically recognize and be activated by EpCAM-positive cells.

### 3.3. Cytotoxicity of EpCAM-CAR-NK-92 Cells In Vitro

**Figure 3:**
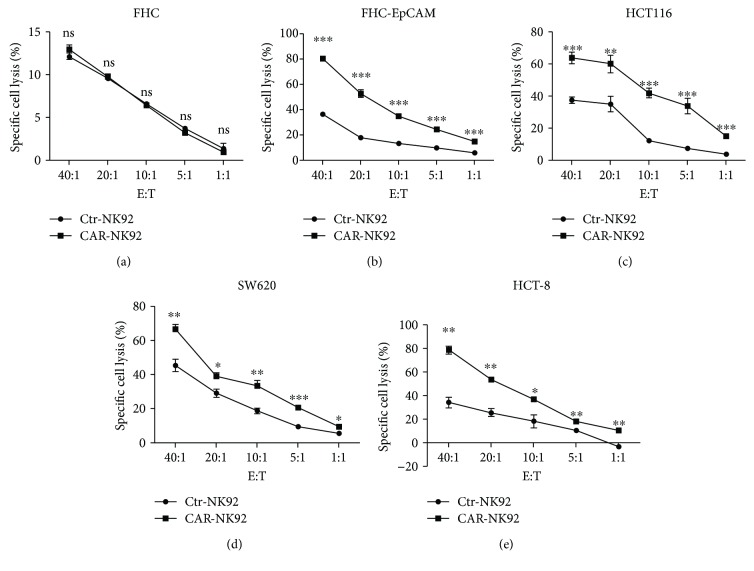
Specific cytotoxicity exhibited by EpCAM-specific CAR-NK-92 cells against EpCAM-positive target cells. The cytotoxic activity of CAR-NK-92 and Ctrl-NK-92 cells against EpCAM-negative or EpCAM-positive cells was determined using a 4-hour lactate dehydrogenase (LDH) release assay in a dose-dependent manner. ^∗^*p* < 0.05; ^∗∗^*p* < 0.01; ^∗∗∗^*p* < 0.001; ns: not significant.

To evaluate the cytotoxicity of the CAR-NK-92 cells against colorectal cancer cells, we performed dose-dependent LDH release assays. As shown in Figure 3, compared with Ctrl-NK-92 cells, CAR-NK-92 cells showed stronger killing activity against EpCAM-positive FHC-EpCAM, HCT116, SW620, and HCT-8 cells at E : T ratios of 40 : 1, 20 : 1, 10 : 1, 5 : 1, and 1 : 1. However, the cytotoxicity difference between Ctrl-NK-92 and CAR-NK-92 against EpCAM-negative FHC cells was not significant. Additionally, the cytotoxicity of CAR-NK-92 cells against EpCAM-positive colorectal cancer cells was positively correlated with the E : T ratios. These results further demonstrated that the CAR-NK-92 cells could specifically recognize and kill EpCAM-positive colorectal cancer cells.

### 3.4. Efficacy of Combination Therapy with EpCAM-Specific CAR-NK-92 Cells and Regorafenib In Vivo

**Figure 4:**
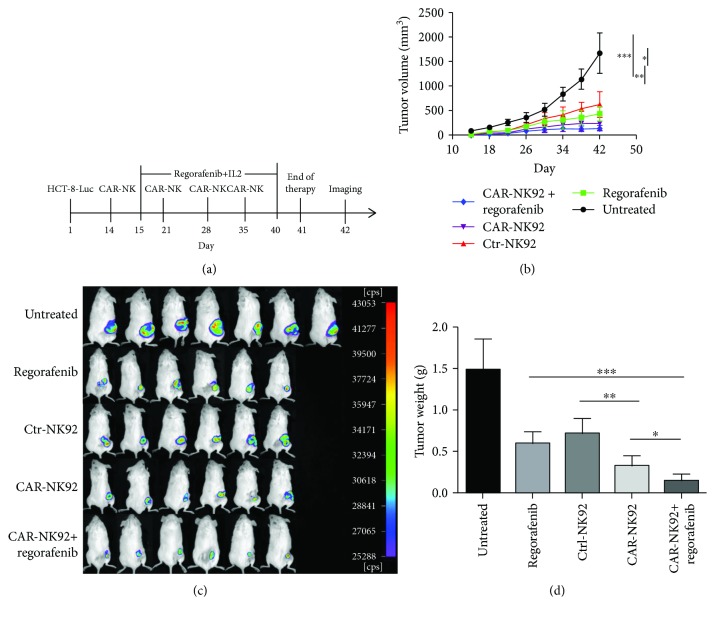
Therapeutic efficacy of EpCAM-specific CAR-NK-92 cells combined with regorafenib for human colorectal cancer xenografts established with HCT-8 cells. (a) Schematic diagram showing the treatment program of the mice. (b) The tumor growth curves during the experiment. (c) Luminescence images showing the tumor size at the end of the treatment. (d) Tumor weight at the end of treatment. ^∗^*p* < 0.05; ^∗∗^*p* < 0.01; ^∗∗∗^*p* < 0.001.

We evaluated the antitumor activity of Ctrl-NK-92 cells, CAR-NK-92 cells, regorafenib, and CAR-NK-92 cells plus regorafenib in NOD/SCID mice with subcutaneous xenograft models established with HCT-8-Luc. The treatment program of the mice is shown in Figure 4(a) and described in Materials and Methods. As shown in Figures 4(b)–4(d), Ctrl-NK-92 cells, CAR-NK-92 cells, or regorafenib treatment alone significantly reduced the growth rate of HCT-8-Luc tumors compared to the untreated group. In addition, treatment with EpCAM-specific CAR-NK-92 cells significantly suppressed tumor growth compared with the Ctrl-NK-92 cells. These results demonstrated the EpCAM-specific killing effect of the CAR-NK-92 cells on the HCT-8-Luc tumors. Furthermore, the combination of the CAR-NK-92 cells and regorafenib significantly reduced the growth rate of HCT-8-Luc tumors compared to regorafenib or CAR-NK-92 cell alone. The tumors of the mice that received CAR-NK-92 cells plus regorafenib treatment were almost eradicated. The values of the tumor volumes (Figure 4(b)), in vivo imaging (Figure 4(c)), and tumor weights (Figure 4(d)) were concordant. These results further demonstrated that the EpCAM-specific CAR-NK-92 cells could specifically recognize and kill EpCAM-positive colorectal cancer cells and that the CAR-NK-92 cells and regorafenib have synergistic antitumor effects against EpCAM-positive colorectal cancers.

### 3.5. In Vivo Persistence of NK-92 Cells

**Figure 5:**
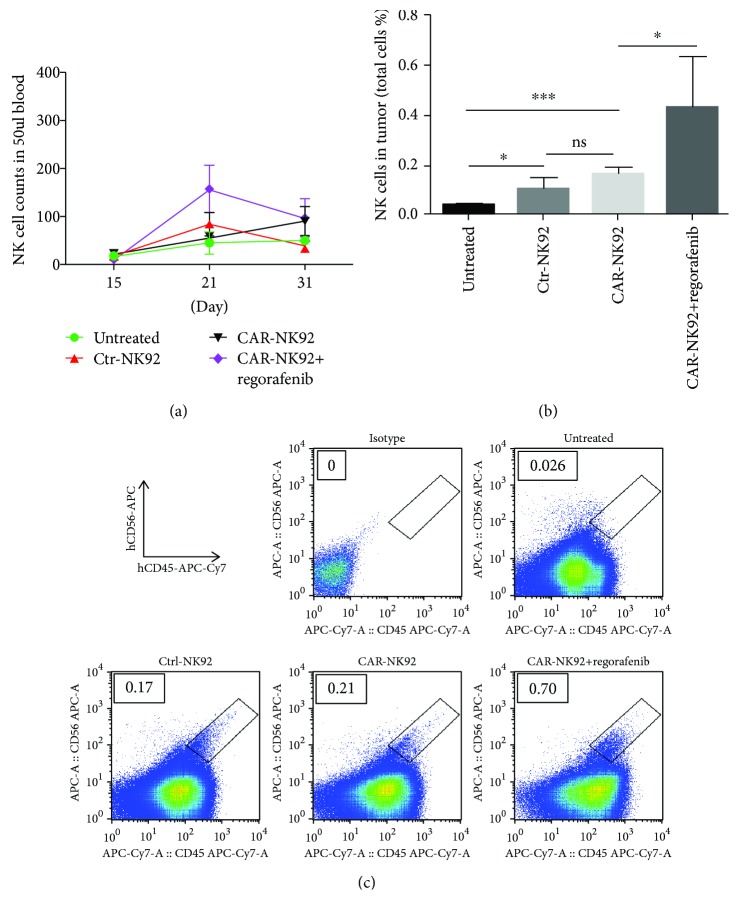
Persistence of NK-92 cells *in vivo*. (a) Continuous persistence of NK-92 cells in the blood of mice by FACS. (b) The quantitative analysis results of NK-92 cells in tumors by FACS. (c) Representative results of NK-92 cells in tumors shown in (b). NK-92 cells were stained with APC-conjugated anti-hCD56 and APC-Cy7-conjugated anti-hCD45 antibodies in all experiments. ^∗^*p* < 0.05; ^∗∗∗^*p* < 0.001; ns: not significant.

To assess the *in vivo* persistence of NK-92 cells, the blood was collected from the tail vein of the mice on days 15, 21, and 31. NK-92 cells in the blood were detected by FACS assays. As shown in Figure 5(a), on day 15, only one day after first transfusion of NK-92 cells, very few NK-92 cells were detected; however, on days 21 and 31, NK-92 cells increased in the CAR-NK-92 and CAR-NK-92 + regorafenib groups, although there was no significant difference between the groups. To assess the homing and persistence ability of the CAR-NK-92 cells, tumor tissues were made into single-cell suspensions. The single-cell suspensions were stained and detected by FACS assays. As shown in Figures 5(b) and 5(c), human NK-92 cells were detectable in tumors derived from mice treated with Ctrl-NK92, CAR-NK-92, and CAR-NK-92 + regorafenib. Furthermore, there were more NK-92 cells in the CAR-NK-92 + regorafenib group than those in the CAR-NK-92 group. Taken together, these data demonstrated that the transfused NK-92 cells can survive and home into tumors *in vivo* and that regorafenib can improve the infiltration of NK-92 cells into tumors.

## 4. Discussion

T cells expressing second-generation EpCAM-specific CARs with the intracellular signaling domain of CD28 were reported to treat prostate cancers in mouse models [42–44]. Ang et al. developed a third-generation EpCAM-specific CAR with intracellular signaling domains of CD28 and 4-1BB. The CAR-modified T cells showed significant efficacy in a mouse model with peritoneal carcinomatosis [10]. NK-92 cells expressing a first-generation EpCAM-specific CAR or coexpressing IL-15 and a second-generation EpCAM-specific CAR with intracellular signaling domains of CD28 were also developed to treat prostate cancer in mouse models [45, 46]. In this study, for the first time, we constructed a second-generation EpCAM-specific CAR with a scFv (MOC31) and the intracellular signaling domain of the costimulation molecule 4-1BB. Our results showed that the CAR-modified NK-92 cells can specifically kill EpCAM-positive colorectal cancer cells. Furthermore, the CAR-NK-92 cells showed significant efficacy against human colorectal cancer xenografts in mice.

Solid tumors, unlike hematological malignancies, have a complex immune microenvironment. Infiltration obstacles and immune suppression of the tumor microenvironment are two important factors that caused the limited efficacy of CAR-T and CAR-NK against solid tumors. Combinations with other treatments, including chemotherapy, radiotherapy, and immune checkpoint inhibitors, will be a promising strategy to improve the efficacy of CAR-T or CAR-NK cells on solid tumors [26]. In this study, we reported a novel regimen, combination therapy with EpCAM-specific CAR-NK-92 cells and regorafenib, to treat colorectal cancer in mouse models. Our results demonstrated that combination with regorafenib can enhance the therapeutic efficacy of the EpCAM-specific CAR-NK-92 cells on colorectal cancer.

A randomized, double-blind clinical trial of regorafenib showed an overall survival of 8.8 months. It is clinically meaningful. However, grade 3 or 4 treatment-related adverse events occurred in 54% of patients [47]. The most common adverse events related to regorafenib in the clinical trial included hand-foot skin reaction, fatigue, diarrhea, hypertension, and rash/desquamation [47, 48]. Dose modifications are an important strategy for managing regorafenib-related side effects. Many patients will need a dose reduction and can continue on therapy without a detrimental effect on their quality of life [47]. The dose of regorafenib we used in this study is lower than the clinical dose. Given a typical human weight of 60 kg, the clinical dose range of 160 mg daily (metastatic colorectal cancer) converts to a human dose of 2.7 mg per kg daily. To convert the human dose into mouse dose, we calculated 2.7 mg per kg × 12.3 = 33.2 mg per kg daily in mouse (the conversion factor 12.3 can be found in FDA guidance at http://www.fda.gov/downloads/Drugs/.../Guidances/UCM078932 and in [39]). Therefore, our regorafenib dose in mice approximates one-third of the approved dose for treating metastatic colorectal cancer. Side effects are one of the important problems caused by chemotherapy drugs for cancer treatment. The novel regimen we reported here may provide a strategy to improve the therapeutic efficacy and decrease the side effects of regorafenib.

To investigate whether regorafenib enhances the antitumor efficacy of EpCAM-specific CAR-NK-92 cells by changing the expression of cytotoxicity-related molecules in colorectal cancer cells, we detected the expression changes of EpCAM, Fas, MicA/B, HLA-A2, PD-L1, and DR4 in CRC cell lines HCT116, SW620, and HT29 after treatment with regorafenib at concentrations of 10 nM for 24 h. We observed that only regorafenib treatment significantly increased the expression level of Fas in SW620 cells and DR4 in HT29 cells and decreased the expression level of PD-L1 in SW620 cells. Because expression changes of these molecules were not consistent in the three CRC cell lines, the data are not shown here.

Several TKIs (sunitinib [49], cabozantinib [50], and doxorubicin [30]) were reported to modulate the tumor microenvironment and promote antitumor immunity. The overall mechanisms are as follows: (1) enhancement of the infiltration of T cells and NK cells by upregulating T/NK cell-related adhesion molecules and chemokines; (2) improvement of the proliferation and function of T/NK cells by downregulating immune suppressive cell subsets (MDSCs, Tregs, TAMs, etc.) and cytokines (IL-10, TGF-*β*, etc.). In this study, in addition to increasing the therapeutic effects of EpCAM-CAR-NK-92 cells through these mechanisms, regorafenib may also improve the function of EpCAM-CAR-NK-92 cells by improving tumor vasculature through its tyrosine kinase inhibition function. Of course, these speculated mechanisms need further confirmation.

## 5. Conclusions

We constructed a second-generation CAR against the tumor-associated antigen EpCAM. Our results showed that the EpCAM-specific CAR-NK-92 cells have a high potential to kill CRC cells, and combination with regorafenib can enhance the effects of the CAR-NK-92 cells against CRC mouse models. The current study is based on the NK-92 cell line. Future analysis of this regimen can also be expanded to autologous or allogeneic primary NK or T cells. This study provides a novel strategy to treat EpCAM-positive CRC.

## Data Availability

The data used to support the findings of this study are available from the corresponding author upon request.

## References

[1] Arnold M., Sierra M. S., Laversanne M., Soerjomataram I., Jemal A., Bray F. (2017). Global patterns and trends in colorectal cancer incidence and mortality. *Gut*.

[2] Hu T., Li Z., Gao C. Y., Cho C. H. (2016). Mechanisms of drug resistance in colon cancer and its therapeutic strategies. *World Journal of Gastroenterology*.

[3] Louis C. U., Savoldo B., Dotti G. (2011). Antitumor activity and long-term fate of chimeric antigen receptor-positive T cells in patients with neuroblastoma. *Blood*.

[4] Pule M. A., Savoldo B., Myers G. D. (2008). Virus-specific T cells engineered to coexpress tumor-specific receptors: persistence and antitumor activity in individuals with neuroblastoma. *Nature Medicine*.

[5] Brown C. E., Alizadeh D., Starr R. (2016). Regression of glioblastoma after chimeric antigen receptor T-cell therapy. *The New England Journal of Medicine*.

[6] Junghans R. P., Ma Q., Rathore R. (2016). Phase I trial of anti-PSMA designer CAR-T cells in prostate cancer: possible role for interacting interleukin 2-T cell pharmacodynamics as a determinant of clinical response. *Prostate*.

[7] Tamada K., Geng D., Sakoda Y. (2012). Redirecting gene-modified T cells toward various cancer types using tagged antibodies. *Clinical Cancer Research*.

[8] Schlimper C., Hombach A. A., Abken H., Schmidt-Wolf I. G. H. (2012). Improved activation toward primary colorectal cancer cells by antigen-specific targeting autologous cytokine-induced killer cells. *Clinical and Developmental Immunology*.

[9] Daly T., Royal R. E., Kershaw M. H. (2000). Recognition of human colon cancer by T cells transduced with a chimeric receptor gene. *Cancer Gene Therapy*.

[10] Ang W. X., Li Z., Chi Z. (2017). Intraperitoneal immunotherapy with T cells stably and transiently expressing anti-EpCAM CAR in xenograft models of peritoneal carcinomatosis. *Oncotarget*.

[11] Morgan R. A., Yang J. C., Kitano M., Dudley M. E., Laurencot C. M., Rosenberg S. A. (2010). Case report of a serious adverse event following the administration of T cells transduced with a chimeric antigen receptor recognizing ERBB2. *Molecular Therapy*.

[12] Hege K. M., Bergsland E. K., Fisher G. A. (2017). Safety, tumor trafficking and immunogenicity of chimeric antigen receptor (CAR)-T cells specific for TAG-72 in colorectal cancer. *Journal for ImmunoTherapy of Cancer*.

[13] Zhang C., Wang Z., Yang Z. (2017). Phase I escalating-dose trial of CAR-T therapy targeting CEA+ metastatic colorectal cancers. *Molecular Therapy*.

[14] Thistlethwaite F. C., Gilham D. E., Guest R. D. (2017). The clinical efficacy of first-generation carcinoembryonic antigen (CEACAM5)-specific CAR T cells is limited by poor persistence and transient pre-conditioning-dependent respiratory toxicity. *Cancer Immunology, Immunotherapy*.

[15] Li Y., Duo Y., Bao S. (2017). EpCAM aptamer-functionalized polydopamine-coated mesoporous silica nanoparticles loaded with DM1 for targeted therapy in colorectal cancer. *International Journal of Nanomedicine*.

[16] Hao H., Zhen Y., Wang Z., Chen F., Xie X. (2013). A novel therapeutic drug for colon cancer: EpCAM scFv-truncated protamine (tp)-siRNA. *Cell Biology International*.

[17] Liao M. Y., Lai J. K., Kuo M. Y. (2015). An anti-EpCAM antibody EpAb2-6 for the treatment of colon cancer. *Oncotarget*.

[18] Pietra G., Vitale C., Pende D. (2016). Human natural killer cells: news in the therapy of solid tumors and high-risk leukemias. *Cancer Immunology, Immunotherapy*.

[19] Gong J. H., Maki G., Klingemann H. G. (1994). Characterization of a human cell line (NK-92) with phenotypical and functional characteristics of activated natural killer cells. *Leukemia*.

[20] Geller M. A., Miller J. S. (2011). Use of allogeneic NK cells for cancer immunotherapy. *Immunotherapy*.

[21] Arai S., Meagher R., Swearingen M. (2008). Infusion of the allogeneic cell line NK-92 in patients with advanced renal cell cancer or melanoma: a phase I trial. *Cytotherapy*.

[22] Zhang Q., Tian K., Xu J. (2017). Synergistic effects of cabozantinib and EGFR-specific CAR-NK-92 cells in renal cell carcinoma. *Journal of Immunology Research*.

[23] Munn D. H., Sharma M. D., Johnson T. S., Rodriguez P. (2017). IDO, PTEN-expressing Tregs and control of antigen-presentation in the murine tumor microenvironment. *Cancer Immunology, Immunotherapy*.

[24] Goswami K. K., Ghosh T., Ghosh S., Sarkar M., Bose A., Baral R. (2017). Tumor promoting role of anti-tumor macrophages in tumor microenvironment. *Cellular Immunology*.

[25] Scarfo I., Maus M. V. (2017). Current approaches to increase CAR T cell potency in solid tumors: targeting the tumor microenvironment. *Journal for ImmunoTherapy of Cancer*.

[26] Xu J., Tian K., Zhang H. (2017). Chimeric antigen receptor-T cell therapy for solid tumors require new clinical regimens. *Expert Review of Anticancer Therapy*.

[27] Huynh H., Ong R., Zopf D. (2015). Antitumor activity of the multikinase inhibitor regorafenib in patient-derived xenograft models of gastric cancer. *Journal of Experimental & Clinical Cancer Research*.

[28] Takigawa H., Kitadai Y., Shinagawa K. (2016). Multikinase inhibitor regorafenib inhibits the growth and metastasis of colon cancer with abundant stroma. *Cancer Science*.

[29] Hu J., Zhu S., Xia X., Zhang L., Kleinerman E. S., Li S. (2014). CD8+T cell-specific induction of NKG2D receptor by doxorubicin plus interleukin-12 and its contribution to CD8+T cell accumulation in tumors. *Molecular Cancer*.

[30] Alizadeh D., Trad M., Hanke N. T. (2014). Doxorubicin eliminates myeloid-derived suppressor cells and enhances the efficacy of adoptive T-cell transfer in breast cancer. *Cancer Research*.

[31] Ko J. S., Zea A. H., Rini B. I. (2009). Sunitinib mediates reversal of myeloid-derived suppressor cell accumulation in renal cell carcinoma patients. *Clinical Cancer Research*.

[32] Finke J. H., Rini B., Ireland J. (2008). Sunitinib reverses type-1 immune suppression and decreases T-regulatory cells in renal cell carcinoma patients. *Clinical Cancer Research*.

[33] Busse A., Asemissen A. M., Nonnenmacher A. (2011). Immunomodulatory effects of sorafenib on peripheral immune effector cells in metastatic renal cell carcinoma. *European Journal of Cancer*.

[34] Cabrera R., Ararat M., Xu Y. (2013). Immune modulation of effector CD4+ and regulatory T cell function by sorafenib in patients with hepatocellular carcinoma. *Cancer Immunology, Immunotherapy*.

[35] Suzuki E., Kapoor V., Jassar A. S., Kaiser L. R., Albelda S. M. (2005). Gemcitabine selectively eliminates splenic Gr-1+/CD11b+ myeloid suppressor cells in tumor-bearing animals and enhances antitumor immune activity. *Clinical Cancer Research*.

[36] Bunt S. K., Mohr A. M., Bailey J. M., Grandgenett P. M., Hollingsworth M. A. (2013). Rosiglitazone and gemcitabine in combination reduces immune suppression and modulates T cell populations in pancreatic cancer. *Cancer Immunology, Immunotherapy*.

[37] Homma Y., Taniguchi K., Nakazawa M. (2014). Changes in the immune cell population and cell proliferation in peripheral blood after gemcitabine-based chemotherapy for pancreatic cancer. *Clinical & Translational Oncology*.

[38] Hsu F. T., Chen T. C., Chuang H. Y., Chang Y. F., Hwang J. J. (2015). Enhancement of adoptive T cell transfer with single low dose pretreatment of doxorubicin or paclitaxel in mice. *Oncotarget*.

[39] Matsushita H., Enomoto Y., Kume H. (2014). A pilot study of autologous tumor lysate-loaded dendritic cell vaccination combined with sunitinib for metastatic renal cell carcinoma. *Journal for ImmunoTherapy of Cancer*.

[40] Takahara A., Koido S., Ito M. (2011). Gemcitabine enhances Wilms’ tumor gene WT1 expression and sensitizes human pancreatic cancer cells with WT1-specific T-cell-mediated antitumor immune response. *Cancer Immunology, Immunotherapy*.

[41] Daudigeos-Dubus E., le Dret L., Lanvers-Kaminsky C. (2015). Regorafenib: antitumor activity upon mono and combination therapy in preclinical pediatric malignancy models. *PLoS One*.

[42] Wu Y., Deng Z., Tang Y., Zhang S., Zhang Y. Q. (2015). Over-expressing Akt in T cells to resist tumor immunosuppression and increase anti-tumor activity. *BMC Cancer*.

[43] Shirasu N., Yamada H., Shibaguchi H., Kuroki M., Kuroki M. (2012). Molecular characterization of a fully human chimeric T-cell antigen receptor for tumor-associated antigen EpCAM. *Journal of Biomedicine and Biotechnology*.

[44] Deng Z., Wu Y., Ma W., Zhang S., Zhang Y. Q. (2015). Adoptive T-cell therapy of prostate cancer targeting the cancer stem cell antigen EpCAM. *BMC Immunology*.

[45] Sahm C., Schonfeld K., Wels W. S. (2012). Expression of IL-15 in NK cells results in rapid enrichment and selective cytotoxicity of gene-modified effectors that carry a tumor-specific antigen receptor. *Cancer Immunology, Immunotherapy*.

[46] Meier R., Golovko D., Tavri S. (2011). Depicting adoptive immunotherapy for prostate cancer in an animal model with magnetic resonance imaging. *Magnetic Resonance in Medicine*.

[47] Melosky B. (2016). Meeting an unmet need in metastatic colorectal carcinoma with regorafenib. *Asia-Pacific Journal of Oncology Nursing*.

[48] Sacré A., Lanthier N., Dano H. (2016). Regorafenib induced severe toxic hepatitis: characterization and discussion. *Liver International*.

[49] Ozao-Choy J., Ma G., Kao J. (2009). The novel role of tyrosine kinase inhibitor in the reversal of immune suppression and modulation of tumor microenvironment for immune-based cancer therapies. *Cancer Research*.

[50] Patnaik A., Swanson K. D., Csizmadia E. (2017). Cabozantinib eradicates advanced murine prostate cancer by activating antitumor innate immunity. *Cancer Discovery*.

