# Corrigendum to “Combination Therapy with EpCAM-CAR-NK-92 Cells and Regorafenib against Human Colorectal Cancer Models”

**DOI:** 10.1155/2019/2070562

**Published:** 2019-03-27

**Authors:** Qing Zhang, Haixu Zhang, Jiage Ding, Hongyan Liu, Huizhong Li, Hailong Li, Mengmeng Lu, Yangna Miao, Liantao Li, Junnian Zheng

**Affiliations:** ^1^Cancer Institute, Xuzhou Medical University, Xuzhou, Jiangsu 221002, China; ^2^The Affiliated Aoyang Hospital of Jiangsu University, Zhangjiagang, Jiangsu 215600, China; ^3^Jiangsu Center for the Collaboration and Innovation of Cancer Biotherapy, Xuzhou Medical University, Xuzhou, Jiangsu 221002, China

In the article titled “Combination Therapy with EpCAM-CAR-NK-92 Cells and Regorafenib against Human Colorectal Cancer Models” [1], there was a typographical error in Figure 4(b) where the label of the statistical difference symbol should be corrected as follows:

## Figures and Tables

**Figure 1:**
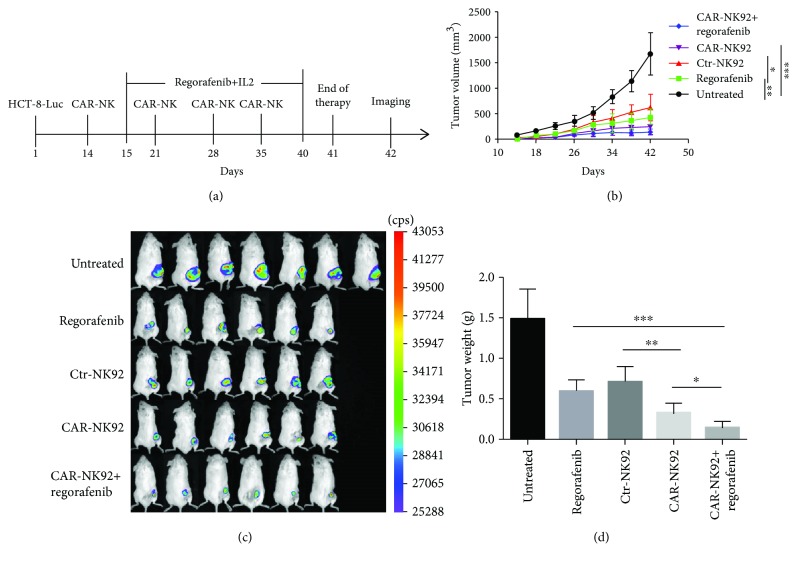
Therapeutic efficacy of EpCAM-specific CAR-NK-92 cells combined with regorafenib for human colorectal cancer xenografts established with HCT-8 cells. (a) Schematic diagram showing the treatment program of the mice. (b) The tumor growth curves during the experiment. (c) Luminescence images showing the tumor size at the end of the treatment. (d) Tumor weight at the end of treatment. ^∗^*p* < 0.05; ^∗∗^*p* < 0.01; ^∗∗∗^*p* < 0.001.
